# Artificial Immune System–Negative Selection Classification Algorithm (NSCA) for Four Class Electroencephalogram (EEG) Signals

**DOI:** 10.3389/fnhum.2018.00439

**Published:** 2018-11-20

**Authors:** Nasir Rashid, Javaid Iqbal, Fahad Mahmood, Anam Abid, Umar S. Khan, Mohsin I. Tiwana

**Affiliations:** ^1^Department of Mechatronics Engineering, National University of Sciences & Technology, Islamabad, Pakistan; ^2^Department of Mechatronics Engineering at the University of Engineering and Technology, Peshawar, Pakistan

**Keywords:** brain computer interface (BCI), artificial immune system (AIS), staked auto-encoder, mel frequency cepstral coefficients (MFCC), electroencephalogram, genetic algorithm

## Abstract

Artificial immune systems (AIS) are intelligent algorithms derived from the principles inspired by the human immune system. In this study, electroencephalography (EEG) signals for four distinct motor movements of human limbs are detected and classified using a negative selection classification algorithm (NSCA). For this study, a widely studied open source EEG signal database (BCI IV–Graz dataset 2a, comprising nine subjects) has been used. Mel frequency cepstral coefficients (MFCCs) are extracted as selected features from recorded EEG signals. Dimensionality reduction of data is carried out by applying two hidden layered stacked auto-encoder. Genetic algorithm (GA) optimized detectors (artificial lymphocytes) are trained using negative selection algorithm (NSA) for detection and classification of four motor movements. The trained detectors consist of four sets of detectors, each set is trained for detection and classification of one of the four movements from the other three movements. The optimized radius of detector is small enough not to mis-detect the sample. Euclidean distance of each detector with every training dataset sample is taken and compared with the optimized radius of the detector as a nonself detector. Our proposed approach achieved a mean classification accuracy of 86.39% for limb movements over nine subjects with a maximum individual subject classification accuracy of 97.5% for subject number eight.

## Introduction

A brain computer interface (BCI) provides a communication channel between the human brain and external devices. This interface is generally composed of a signal processing device, a set of non-invasive sensors and an external device. The electroencephalogram (EEG) based BCI system is generally composed of three processes namely, signal preprocessing, feature extraction, and classification. The signal preprocessing step is performed to enhance the acquired signal by removing baseline noise and selecting a band frequency of interest. The feature extraction step is mainly employed to extract meaningful information in the form of features from raw EEG signals. The last step of classification is performed to encode the specific features into meaningful information (determining the class) in order to control the external devices. Since the BCI system is independent of muscles and peripheral nerves, it is mainly beneficial for patients who suffer from motor disorders with cognitive disabilities (Wolpaw et al., 2002; Leuthardt et al., 2004; Kronegg et al., 2007; Kachenoura et al., 2008).

Electroencephalogram based brain computer interface systems generally consist of three phases. The initial phase is composed of a training phase, which includes learning and calibration of a classification system, using training data. In the second phase, test data is presented to the calibrated and trained system, which in turn classifies data in the respective class. A third phase may also be added to the system, called an operational phase in which the system performs an online task to recognize brain signals, translating them into computer commands (Lotte, 2014).

Pattern recognition is one of the most vital tasks of the BCI system. In the past, several classification and feature extraction approaches have been proposed, suitable for motor imagery task recognition. Common spatial pattern (CSP) is one of the most widely used feature extraction approaches (Müller-Gerking et al., 1999; Ramoser et al., 2000). Other well-known approaches include principal component analysis (Jolliffe and Cadima, 2016) for dimensionality reduction and independent component analysis (Comon, 1994) for artifact removal. False discovery rate, using a correlation and coherence model as features, have been used for fNIRS data, employing the NIRS brain AnalyzIR toolbox (Santosa et al., 2017, 2018). The time points feature extraction or band power after spatial filtering approach is also employed as a feature extraction technique to deal with non-stationarity in the data for EEG classification in BCI systems (Arvaneh et al., 2013).

Many traditional approaches have been employed for the classification of motor imagery, those include a linear discriminant analysis (LDA) (Hong et al., 2015; Hong and Santosa, 2016) and a quadratic discriminant analysis (QDA) (Fukunaga, 2013), support vector machines (SVM) (Naseer and Hong, 2013) and a Bayesian classifier (Nielsen and Jensen, 2009). The deep belief network approach has also been used for motor imagery EEG classification and achieved comparatively higher accuracy than discriminant classifiers (An et al., 2014). The adaptive LDA/QDA classification approach using band power as a feature extraction technique for motor imagery EEG data has been also employed successfully (Schlögl et al., 2009). An effective application of the Riemannian geometry based SVM kernel and the targeted band-pass covariance as a feature extraction approach for motor imagery has been reported by Barachant et al. (2013). In the context of motor imagery EEG classification, Riemannian geometry is currently utilized for getting more accurate and robust prediction models. Riemannian geometry is logarithmic in nature making it inherently robust to noise signals. In addition, P300 EEG classification using Riemannian geometry and special covariance as a feature extraction approach has been employed efficiently (Mayaud et al., 2016). Another approach for motor imagery EEG classification, using a linear discriminant analysis and a topographic map as feature extraction techniques, has displayed comparative results (Phan and Cichocki, 2010). Kang et al. (2009) employed the CSP and band power for motor imagery classification, utilizing a linear support vector machine. Using surface laplacian as a feature extraction method and employing LDA and Bayesian models as a classifier for motor imagery EEG signals has proved to be an effective method (Jayaram et al., 2016). Lu et al. (2009) performed P300 EEG classification using time points as a feature extraction approach and fisher LDA as a classification approach. Using band power as a feature extraction approach while utilizing the deep belief neural network for classification purposes has also been proposed (Sturm et al., 2016). Tabar and Halici (2016) proposed a combination of the convolutional neural network and the deep belief neural network for motor imagery EEG classification and explored band power as a feature extraction approach.

This research presents a framework for the application of an artificial immune system (AIS) for the detection and classification of four class motor imagery EEG signals. The field of AIS has seen the considerable research direction recently in network traffic flow monitoring and bearing fault detection in motors, however, it has not been utilized in applications involving classification of EEG brain signals. The main contribution of this research is to develop an AIS algorithm inspired by the human immune system for a multiclass classification. For this purpose, we selected a widely used open source EEG signal database (BCI IV–Graz dataset 2a, comprising nine subjects). Mel-frequency cepstral coefficients (MFCCs) have been selected for extracting features from preprocessed EEG signals. Two hidden layers Stacked Auto-Encoder (SAE) is used for dimensionality reduction of data to reduce computational complexity, however, ensuring maximum representation of data based on accuracy. Genetic algorithm (GA) optimized detectors (artificial lymphocytes) are trained using a negative selection approach (NSA) to dectect four motor movements. Four sets of detectors are trained to detect and classify each movement from the other three movements. In the training process, detection and classification accuracies are measured and based on maximum accuracy; the set of detectors is selected and saved for each class training sample. Furthermore, AIS trained detectors are then used to classify test data samples containing all four movements. Our proposed algorithm has shown significant improvements in classification accuracy as compared with recent state of the art approaches.

## Materials and methods

### Section I–Immune system theory

Each human being has a biological immune system (BIS) whose characteristics and complexity vary according to the individual. The most vital function of an immune system is to protect organisms against the attack of foreign agents causing disease, called pathogens. The BIS is an adaptive system that has evolved to provide protection against pathogens. The BIS provides this protection through sophisticated pattern recognition and response mechanisms and depending on the type of invaders, the damage it can cause and the way it enters the body. The BIS uses various response mechanisms either to destroy the invader or to neutralize its effect (Dasgupta, 2006). Proteins found on the surface of pathogens are called antigens and are unique to that pathogen. Like pathogens, our own body tissues also contain antigens known as self-antigens and those found on the surface of pathogens are called nonself antigens. The process of discrimination between self-antigens and nonself antigens is known as self/nonself discrimination. The two BIS organs, the thymus gland and bone marrow, are responsible for the maturation and production of immune cells called lymphocytes. Lymphocytes are white blood cells responsible for the recognition of nonself antigens. Lymphocytes that are generated within the thymus are known as T-cells, while lymphocytes generated within the bone marrow are known as B-cells (Warner, 1974).

The BIS can be broadly classified into specific and nonspecific immunity. Nonspecific immunity is accredited to generalized defense without identifying specific types of pathogens. This nonspecific immune system is known as the innate immune system because the human body is naturally capable of the general ability to attack and destroy specific types of microbes. The specific immune system is developed for the defense of targeted pathogens only. The backup immune system also constitutes the overall immune system in which the immunity or defense is artificially acquired under special cases (Janeway et al., 2005).

The innate immune system is composed of defense layers against foreign particles, viruses, parasites, and bacteria. This is a generalized and nonspecific immune system providing generic defense mechanisms to nullify the influence of intruders against body cells. The innate immune system targets anything that is identified as nonself.

The specific immune system is further classified as cell-mediated immunity or antibody-mediated immunity. An antibody is a blood protein produced in response to a specific antigen. Antibodies combine with substances that the body recognizes as nonself, such as bacteria, viruses, etc. This system is composed of antibodies that attack and destroy antigens and foreign body particles. Cell mediated immunity is composed of lymphocytes, which play a vital role as mediators of counteracting antigens. Lymphocytes are further classified as B-cells and T-cells, produced in the bone marrow and thymus, respectively. In addition, T and B cells have specific receptors that are matched with only one type of antigen. Furthermore, T-cells help the B-cells in producing special Y-shaped proteins (antibodies). Each group of antibodies has the capability to grab a specific type of invader. These antibodies stick with the body of antigens by matching with the molecules on the surface of antigens. These antibodies eventually destroy the foreign antigen.

The backup immune system artificially influences the immune response. This immune system differs from the naturally acquired immunity (specific immune system or innate immune system) in a way that it is artificially acquired through vaccination. Sometimes there is a need for immunosuppression, which is the reduction in efficacy or activation of other parts of the immune system. Immunosuppression is performed intentionally with the help of vaccines in case of an organ transplant. This is done to avoid a false attack on nonself antigens.

### Section II–Artificial immune system (AIS) theory

Artificial immune system is a new and emerging soft computing method. In past decades, researchers have found various engineering and computational solutions through AIS. An algorithm known as the artificial immune recognition system (AIRS) was developed by Goodman et al. (2002) and tested against Kohenon's learning vector quantization (LVQ) algorithm and the k-nearest neighbors (kNN) algorithm. In addition, AIRS proved to be more efficient in terms of binary classification accuracy. The algorithm was further developed for multiclass classification through several stages to train a population of data points (Watkins and Boggess, 2002). Furthermore, AIS is described as an adaptive system that imitates immune functions and models principles to solve difficult problems (Castro et al., 2002). In this paper, a negative selection principle is used for the detection and classification of motor imagery.

Negative selection algorithm (NSA): It is imperative for the BIS to distinguish between foreign cells and endemic cells to function properly. Malfunctioning of the distinction between self and nonself, can lead to an autoimmune disease. Cells with receptors are able to recognize self-cells and are known as auto-reactive cells. The elimination of any self-reactive cell characterizes the negative selection concept (Nino and Dasgupta, 2008). Negative selection provides control to T and B lymphocytes eliminating potential autoreactive cells during development. The thymus gland is responsible for the maturation of T cells, and their negative selection occurs inside it; only those T cells survive that do not recognize self-cells (Timmis et al., 2004). The first optimization of the NSA was done in 2009, and a population of antibodies was compressed for faster training and classification (Elberfeld and Textor, 2009). The research was further enhanced to show that the NSA can be simulated without generating any set of antibodies, however, this simulation was not implemented (Liśkiewicz and Textor, 2010). A method to optimize the NSA using a division of the feature space with “neighborhood” for both antibodies and samples was proposed (Wang et al., 2011; Textor, 2012). They introduced a method to improve matching operations between detectors and samples, improving performance in high dimensions. The NSA has the characteristics of pattern recognition and has been developed on the basis of the negative selection of T cells in the thymus. The NSA is executed in two phases, which can be defined as follows (Forrest et al., 1994):

- In the first phase, known as the censoring phase, two different data sets are defined as P and C. Set P consists of a training set, which contains self-patterns, and set C consists of arrays of randomly generated numbers with the same number of elements in one array as in set P. The affinity between each array of set C is computed with each array of set P. If the affinity between the arrays of set C and at least one array of set P is greater than the threshold, it is rejected (negatively selected). Alternatively, it will be stored in the set R. This phase, is called the censoring phase, and a set of nonself detectors R is achieved.
- In the second phase, called the monitoring phase, set R is used to detect a nonself pattern from test set P^*^. The affinity between each array of set R and each array of set P^*^ is computed, and nonself patterns from the test set are identified.

**Figure 1:**
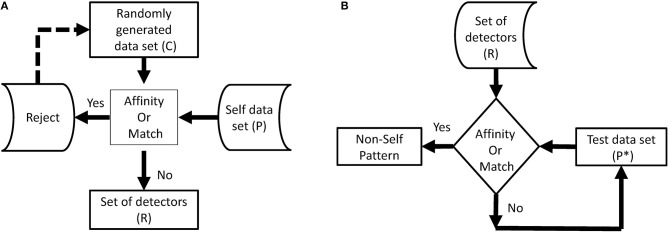
Flowchart of two phases of NSA. **(A)** Flowchart–phase-1 NSA. **(B)** Flowchart–phase-2 NSA.

In NSA, the detector set R is referred to as mature T cells that can recognize pathogens (nonself). Figures 1A,B show the flow chart of both phases of NSA. In addition, NSA works on the similar principle of the biological negative selection process, which occurs in the thymus. Furthermore, NSA is a mathematical formulation of the BIS, which detects external invaders and does not react with self-cells.

### Match or affinity measures

In Forrest et al. (1994), NSA was proposed for computer network security tasks, however, in our application, patterns are real valued numbers, which are voltage levels. For our research, we used Euclidean distance, Equation (3), as the measurement of affinity between the data set and detectors. The real valued vectors C (detectors) and P (self-data) can be given as Equations (1,2)

(1) $$C^{k}=\left[c_{1}^{k}\;c_{2}^{k}\;\ldots \ldots \;c_{Q}^{k}\right]$$

where *k* = 1, 2, ……*N*_*c*_

(2) $$P^{i}=\left[p_{1}^{i}\;p_{2}^{i}\;\ldots \ldots \;p_{Q}^{i}\right]$$

where *i* = 1, 2, ……*N*_*p*_

(3) $$dist(C^{k},P^{i})=\sqrt{\sum_{j\;=1}^{Q}{\left|c_{j}^{k}-p_{j}^{i}\right|}^{2}}$$

### Section III–Data set used for study

**Figure 2:**
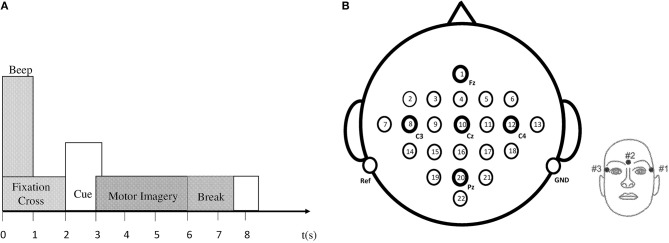
**(A)** Timing pattern of the data acquisition protocol. **(B)** Left: electrode arrangement according to international 10–20 system. Right: electrode placement of three monopolar EOG channels (Brunner et al., 2008).

The EEG data set employed for classification in this research article is taken from the BCI IV–Graz dataset 2a, comprising of nine subjects (Brunner et al., 2008). The BCI data comprises of EEG signals of four imaginary movements of the left hand (class 1), right hand (class 2), both feet (class 3), and tongue (class 4). The subjects were trained to record the data before it was actually recorded. During data acquisition, all subjects sat comfortably on a chair, their eyes fixed on the computer screen, waiting for the appearance of a fixation cross to occur at *t* = 0 s. After 2 s, a cue in the form of an arrow (up, down, left, or right) appeared on the screen along with a fixation cross. Subjects had to imagine motions of the tongue, feet, and left or right hands, upon viewing the arrows (up, down, left, or right) correspondingly. The arrow disappeared after 1.25 s, while the fixation cross remained on the screen. All subjects were required to imagine motor movement tasks according to the cue (arrow) until the fixation cross disappeared from the screen at time *t* = 6 s. Each run consisted of 48 independent trials. Every session consisted of six runs with short breaks accumulating to a total of 288 trials per session. Figure 2A demonstrates the timing diagram of the EEG data acquisition protocol.

Data recording was performed on head-sets with 25 Ag/AgCl electrodes each, set 3.5 cm apart. Twenty two channels provided EEG signals, and three EOG channels (monopolar) were logged at a 250 Hz sampling rate. Figure 2B demonstrates the diagram of electrode montage for the EEG data acquisition. The sampling frequency of acquired EEG was 250 Hz, and further filtering between 0.5 and 100 Hz was carried out by a band-pass filter. The signals were also amplified with an amplifier with a sensitivity of 100 μ*V*. Noise suppression was finally performed with a notch filter operating on a 50 Hz frequency. In our research, we did not use three EOG channels for classification.

**Figure 3:**
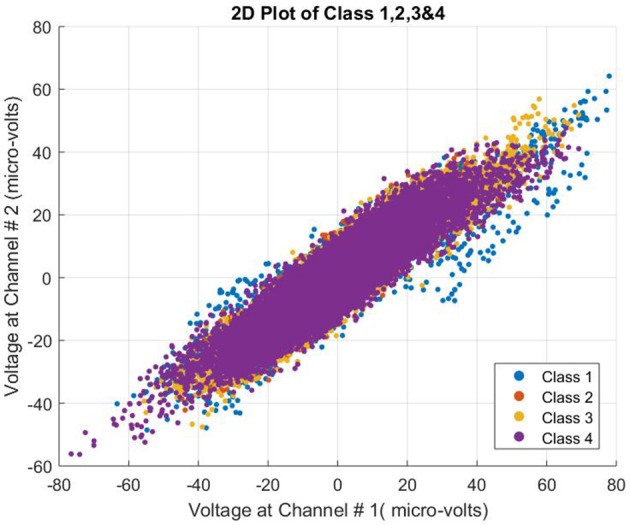
2D plot of all four classes of subject 1.

The data used for this study proved challenging. All four class patterns were intermingled and not easily separable. The complexity of the data can be seen in Figure 3.

**Figure 4:**
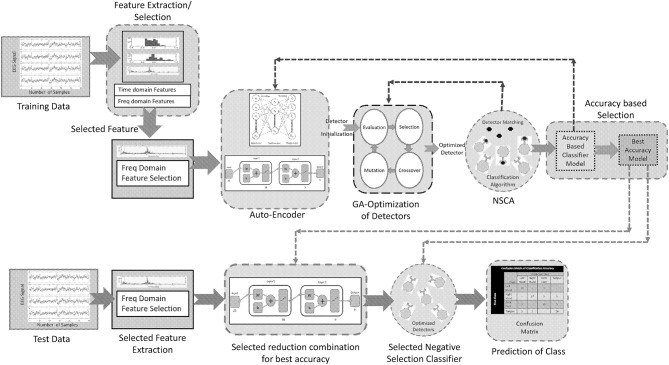
Layout of proposed method.

### Section IV–Proposed scheme

A noninvasive brain signal EEG can provide a pattern of the thought process of a BCI user, which can be interpreted by the use of intelligent algorithms comprising of classifiers. These classifiers can predict the intentions of the user with considerable accuracy, and these predictions can be used for communication with external world. This paper presents an intelligent algorithm that combines preprocessing of a brain signal (stage 1), reduction of dimensionality while keeping the maximum information of the signal (stage 2), and a negative selection classification algorithm (NSCA) as a detection and classification technique (stage 3). Figure 4 shows the layout of the proposed method.

**Figure 6:**
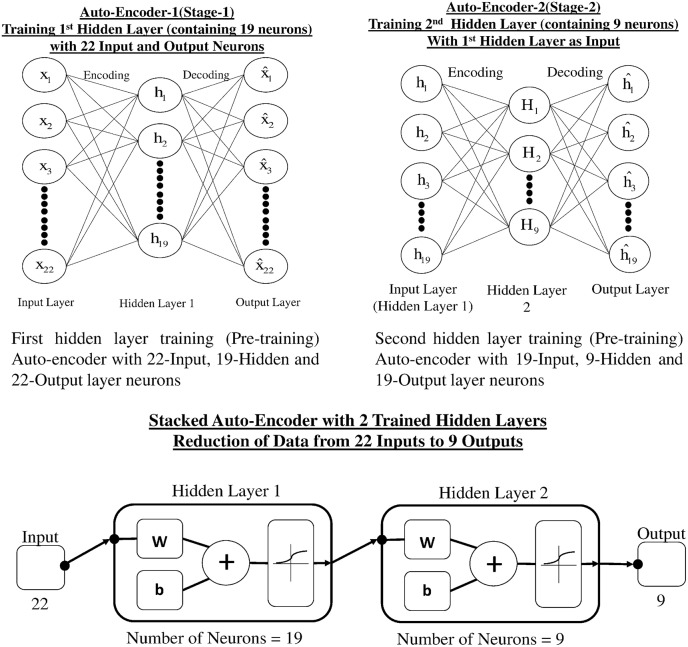
Stacked auto-encoder with two pre-trained hidden layers and final reduction of data.

#### Feature extraction

Electroencephalogram signals are preprocessed by the equipment used for data recording. Frequency ranging from 0.5 to 100 Hz is extracted using a built-in band-pass filter. Line noise has also been removed using a notch filter of 50 Hz. An EEG signal can be represented in many ways. The two most common types of features used to represent an EEG signal are time domain statistical features and frequency domain band power features (Guyon and Elisseeff, 2003). Time domain statistical features are extracted after preprocessing, like band-pass or low-pass filtering and down sampling. These features help classify event related potentials (ERPs) (Blankertz et al., 2011). The frequency domain band power features represent the power (energy) of EEG signals for a given frequency band in a given channel. This energy is averaged over a given time frame (selected for a particular application; Brodu et al., 2011). Time point features are sequences of EEG samples for all channels. These features are typically used to classify ERPs, which lead to temporal variation in EEG signals. These features are used mostly in P300 based BCIs (Breiman, 2001; Lotte, 2014). Band power features are considered the gold standard feature for BCIs, based on motor and mental imagery (Lotte et al., 2018). In our study, we extracted features from both domains as discussed above and the selected feature is used for classification. The time domain statistical features used for this study are given in Table 1.

**Table 1:** Time domain features used in study.

**Feature**	**Mathematical model**
Mean absolute deviation (MAD)	$\text{MAD}=\frac{1}{\text{n}}\sum_{\text{i}=1}^{\text{n}}\left|{\text{s}}_{\text{i}}-\bar{\text{s}}\right|$
Mean value (μ)	$\mu =\frac{1}{n}\sum_{i=1}^{\text{n}}{\text{s}}_{\text{i}}$
Standard deviation (σ)	$\sigma =\frac{1}{\text{n}}\sqrt{\frac{\sum_{i=1}^{\text{n}}{\left({\text{s}}_{\text{i}}-\bar{\text{s}}\right)}^{2}}{\text{n}-1}}$
Variance (σ^2^)	$\sigma^{2}=\frac{1}{\text{n}-1}\sum_{i=1}^{\text{n}}{\left({\text{s}}_{\text{i}}-\bar{\text{s}}\right)}^{2}$
Kurtosis (m_4_)	${\text{m}}_{4}=\frac{1}{\text{n}-1}\sum_{i=1}^{\text{n}}{\left({\text{s}}_{\text{i}}-\bar{\text{s}}\right)}^{4}$

*Where s_i_ is the ith element in the vectors of length n and $\bar{s}$ is the mean value of s*.

Frequency domain features used for this study are MFCCs. In addition, MFCCs are extensively utilized in signal processing owing to their robustness, nonlinear frequency scale, and de-correlated nature (Dharanipragada et al., 2007). In this research article, we have considered the similarity of sound signals to EEG signals using MFCCs as features. Cepstrum can be seen as the rate of change in different spectral bands. In order to use a power spectrum as a feature for representing a signal, the spectrum is first transformed using a mel scale to establish the mel frequency cepstrum. The power cepstrum of a signal *x*[*n*] is calculated by Equation (4).

(4) $$P_{x[n]}={\left|F(log({\left|F\left(x\left[n\right]\right)\right|}^{2})\right|}^{2},$$

where *F* is the Fourier transform. A complex cepstrum of a signal *x*[*n*] is defined by using its Z-transform and is given by Equation (5)

(5) $$C_{x[n]}=Z^{-1}lo\text{g}\left(Z\left[x\left[n\right]\right]\right)$$

The MFCCs are extracted first by framing and windowing the signal followed by taking the Fourier transform. The resultant spectrum magnitude is wrapped by the mel scale and discrete cosine transform (DCT). The log of this spectrum increases the computational efficiency (Kinnunen, 2003; Nasr et al., 2018). The mel scale is defined as a perceptual scale of pitches, and the following Equations (6,7) are used to convert the signal frequency from hertz (Hz) to mel (m).

(6) $$m=2595\log_{10}\left(\frac{f}{100}+1\right)$$

(7) $$m=1127\log_{e}\left(\frac{f}{100}+1\right)$$

The MFCC vector is computed for each frame using short term analysis. In this process, to reduce the blurring effects of the signals, it is pre-emphasized. The pre-emphasis is achieved by applying the first order finite impulse response (FIR) filter of the form (Kinnunen, 2003) to reduce the blurring effect of the signal and is given by Equation (8)

(8) $$H\left(z\right)=1-az^{-1},$$

where 0.9 ≤ *a* ≤ 0.99

The signal is divided into small sections, called frames, and this process is derived from a quasi-stationary nature of signals. However, if these signals are observed as discrete sections over a short duration, then these demonstrate stable characteristics and can be considered stationary (Kinnunen, 2003; Nasr et al., 2018). Frame overlapping helps to avoid loss of information from the signal. To increase the continuity between adjacent frames, a windowing function is applied for each frame. The most common windowing functions are the Hamming and Rectangular window followed by the Blackman and Flattop window. While dealing with time domain cases, the windowing operation can be achieved by multiplying the frame and window function on a point to point basis. The windowing operation corresponds to the convolution between the short term spectrum and the windowing function frequency response. The most commonly used function is the Hamming Window, given in Equation (9), which is defined by Kinnunen (2003); Nasr et al. (2018).

(9) $$w_{H}\left(n\right)=0.54-0.46\cos\left(\frac{2n\pi }{N-1}\right),$$

where *n* = 0, 1, ………….., *N*−1 and *N* is the number of frames the signal has been divided into.

Magnitude spectrum is obtained by computing the discrete fourier transform (DFT) of a windowed frame of the signal. Mathematically DFT is defined as Equation (10)

(10) $$S\left(k\right)=\sum_{n\;=0}^{N-1}s(n)e^{\frac{-j2\pi }{N}kn},$$

where *s*(*n*) is the time sample of a windowed frame. Arithmetically, IDFT (inverse discrete fourier transform) is given by Equation (11) (Kinnunen, 2003; Nasr et al., 2018).

(11) $$S\left(n\right)=\frac{1}{N}\sum_{k=0}^{N-1}S(k)e^{\frac{-j2\pi }{N}kn}$$

Magnitude spectrum is wrapped in the form of frequency in order to transform the spectrum into a mel-frequency scale. Wrapping of mel-frequency is achieved by the mel-filter bank, which contains a band-pass filter as set with constant spacing and bandwidths. The filter bank comprises of one filter for each desired mel-frequency component. Moreover, each filter has a triangular filter band-pass frequency response. The range from zero to nyquist frequency is encompassed with the spread of triangular filters. The recognition accuracy of the system is effected by the number of filters that are to be set according to mel frequency components. In addition, DCT is performed on mel-spectrum logarithm (log) as the last stage, and the resulting amplitudes are called the MFCCs. If the energy of the *m*th mel-filter output is given as $\tilde{S}\left(m\right)$, the MFCCs will be given by Equation (12)

(12) $$c_{j}=\sqrt{\frac{2}{N_{f}}}\sum_{m\;=1}^{N_{f}}log(\tilde{S}(m))\cos\left[\frac{j\pi }{N_{f}}\left(m-0.5\right)\right],$$

where *j* = 0, 1, …………., *j*−1, with *j* being the number of MFCCs, *N*_*f*_is the number of mel-filters and *c*_*j*_ are the MFCCs. As maximum signal information is represented by the first few MFCCs, the number of resulting coefficients is selected between 12 and 20 (El-Samie, 2011). We can castoff the zeroth coefficient as it represents the mean log energy of the frame. For our study, we have chosen 12 MFCCs referred to as static parameters of the frame (Martin et al., 2008). The complete process of MFCC includes windowing, computation of fast fourier transform, computation of log amplitudes of spectrum into mel scale, and computation of discrete cosine transform of mel log amplitudes.

#### Feature selection

Feature extraction is followed by a feature selection step to select a subset of extracted features with potential benefits (Guyon and Elisseeff, 2003). The feature selection step has to be carried out carefully, firstly, some of the extracted features may be redundant or may not be related to the state targeted by BCI. Secondly, the features may be positively correlated with parameters to be optimized by the classifier. Thirdly, from the point of view of knowledge extracted from the signal only a few of the features should actually be related to the targeted states. Fourthly, a model with fewer numbers of feature will give faster predictions for a new sample and at the same time will be computationally efficient and less expensive. Fifthly, storage of data will be reduced. The filter, wrapper, and embedded approaches have been suggested in Kohavi and John (1997).

**Figure 5:**
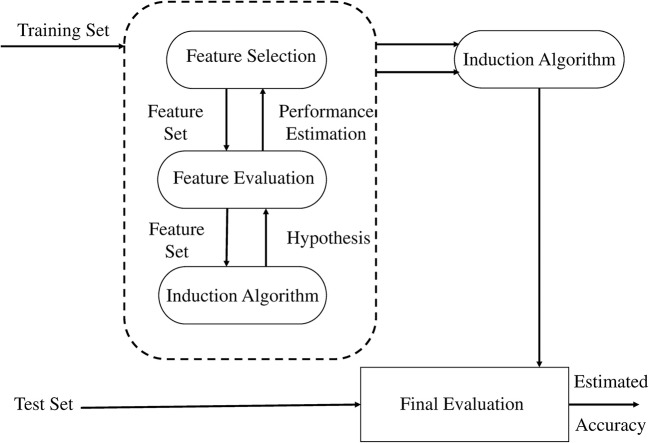
The wrapper approach to feature subset selection. The induction algorithm is used as a “black box” by the subset selection algorithm (Kohavi and John, 1997).

In our study, we adopted the wrapper method for selection of a subset of features (Kohavi and John, 1997). In the wrapper method, the subset is selected and presented to a classifier as an input sample for training, and the resulting performance is observed. If the resulting performance does not meet the criteria, a new subset is selected and the procedure is repeated until the desired criteria is met or maximum performance is achieved. Classifier performance is calculated offline for a pre-recorded training data set, while evaluating the held out data set. However, cross validation can also be used for performance evaluation. We used SVM as a classifier for determining classification accuracy using holdout validation method. The MFCCs are selected as best features for our application. Owing to their regularization property and immunity to curse of dimensionality, SVM are considered most efficient classifiers for BCI applicaitons (Lotte et al., 2007). The wrapper approach as suggested in Kohavi and John's (1997) is shown in Figure 5.

#### Dimensionality reduction

Auto-encoders (AE) are used for learning more about the structure of data without using any class or labels, that is, an unsupervised learning technique. An AE is a single layer neural network, which has one hidden layer and the network tries to reconstruct the input. The common constraint of an AE is called a bottleneck constrain in which the number of neurons in the hidden layer is less than the input and output layer. This constraint makes the network learn a compressed representation of data, which leads to a dimensionality reduction (Rumelhart et al., 1985). Our proposed dimensionality reduction technique uses a two layer stacked auto-encoder. Stacked auto-encoder based pertaining is one of the approaches that can reduce the dimensionality of data and yields less over-fitting. Figure 6A represents the architecture of stage-1 single hidden layer auto-encoder, which carries out the first reduction in data dimension. Figure 6B represents the second phase of reduction of data in which trained hidden layer of stage-1 is used as input to stage-2. Figure 6C shows the reduction of input data (22 channels) in two stages through stacked AE consisting of trained hidden layers of stage-1 and stage-2. The output of two stage stacked AE is reduced to nine channels.

The input $x\in {\left[0,1\right]}^{n_{x}}$ is encoded to a hidden layer as $h\in {\left[0,1\right]}^{n_{h}}$, which represents the vectors of the hidden layer. Now, the hidden layer will be decoded to an output $x\prime \in {\left[0,1\right]}^{n_{x}}$. ***x*′** represents the reconstruction of input by training the hidden layer through minimizing the difference between the two. The model learns to map a feature representation *h* that can reconstruct the input. Equations (13,14) represent the encoding and decoding.

(13) $$h_{j}=sigm(b_{j}+\sum_{i\;=1}^{n_{x}}w_{ij}x_{i})$$

(14) $$x\prime i=sigm(a_{i}+\sum_{j\;=1}^{n_{h}}w\prime_{ij}h_{j})$$

Here, dimensionality of input and hidden layer is given by *n*_*x*_and *n*_*h*_ while $sigm\left(x\right)=\frac{1}{1+e^{-x}}$ is the logistic sigmoid function element wise. $W=\left[w_{ij}\right]\text{ and }W\prime =\left[w\prime_{ij}\right]$ are weight matrices and *a* = [*a*_*i*_] and *b* = [*b*_*j*_] are bias vectors.

The weights of auto-encoder are learned and updated using the Lavenberg-Marquardt back propagation algorithm (Yu and Wilamowski, 2011). Given a training set of *N* input data vectors, each training set *x*^(*n*)^ can be mapped through a hidden representation *h*^(*n*)^ by reconstructing *x*′(*n*). The model parameters Θ = {*W, a, b*} are tuned to minimize the loss function, which is the mean squared reconstruction error over the training set. The mean squared error can be represented by Equation (15)

(15) $$J\left(\theta \right)=\;\frac{1}{N}{\sum_{n=1}^{N}\left\|x^{\left(n\right)}-x\prime (n)\right\|}^{2}$$

There is a chance of calculated weights to increase unboundedly, which may cause over-fitting and computational complexity. To improve generalization on unseen data, a regularization term is added to the encoder. This term is also called weight decay term, and it shrinks the weights in layer 2, which have very small values. The regularizer can also be employed in layer 1, and it can reduce the redundant weight to zero. This gives rise to selective activation of hidden units, which are more useful for the discriminative tasks (Coates et al., 2011). Commonly sparsity cost is calculated, based on cross entropy between the sparsity (average activation) of each unit, ${\hat{\rho }}_{j}=\frac{1}{N}\sum_{n=1}^{n}h_{j}^{\left(n\right)}$, and a user defined sparsity ρ. The term ρ is sparsity parameter and typically set very close to zero. The entire objective function can be given by Equation (16)

(16) $$\begin{array}{l} J(\theta )=\frac{1}{N}\sum_{\text{n}=1}^{N}{\left\|x^{\left(n\right)}-x\prime^{\left(n\right)}\right\|}^{2}+\lambda \|W{\|}^{2}\\ \;+\beta \sum_{\text{j}=1}^{n_{h}}\rho \log\frac{\rho }{{\hat{\rho }}_{\text{j}}}+(1-\rho )\log\frac{\left(1-\rho \right)}{1-{\hat{\rho }}_{\text{j}}} \end{array}$$

Here, λ and β are hyper parameters to tune the effects of weight decay and sparsity cost, respectively. Gradient decent based approach is used to reduce the objective function.

#### GA based optimized negative selection classification algorithm (NSCA)

Proposed selection of features and an optimal reduction in dimensionality of data provides us the most suitable combination for classification of four class motor imagery using the negative selection classification algorithm (NSCA). The reduced feature data is normalized, and the detectors are generated in the reduced low dimensional self-sample space. Otherwise, high dimensional data space can lead to an exponential increase in computational load. Since we have EEG signals pertaining to four classes of human limb movement, we have to generate, optimize, and train four sets of detectors corresponding to each movement class. The detectors are trained using the self-sample (training sample), and the best optimized detector set for the particular class is saved. The selection of the best set of detectors is based on accuracy. As the best sets of detectors for each class are obtained through training (self) samples, our algorithm computes the classification accuracy for the test samples (unknown data) for all classes. For training of detectors, knowledge of only self-sample is given to NCSA, and after the detectors are optimized, the best selected set is used for the classification of the test sample. Classification of motor imagery based four human limb movements using NCSA is a four stage process: (1) Selection of combination of neurons in hidden layers; (2) Detector generation and optimization; (3) Selection of best set of the optimized detectors; and (4) Classification of the test data set.

##### Selection of combination of neuron in hidden layers

In the first stage of dimensionality reduction of selected feature set, a combination of number of neurons is selected according to Huang (2003). In addition, EEG signals are subject specific and possess a large intra-subject diversity of data (Wang et al., 2017), so the selection of reduction of dimensions for each patient is carried out by calculating the detection accuracy of self-samples against nonself samples. A range for number of neurons in first hidden layer (15–19) and second hidden layer (6–10) has been selected. All possible combinations have been evaluated for dimensionality reduction of training sample set and tested on test data set for detection accuracy of self samples. The best combination of number of neurons is selected for dimensionality reduction and subsequently training all the detector sets. Number of detectors has also been varied from 25 to 70 for all combinations of hidden layer neurons. Experimental results have shown greater accuracies in the range of 30 to 40 number of detectors, therefore, the results are shown only for this range of number of detectors and all combinations of hidden layer neurons. The range of 30–40 number of detectors yield optimum result because when we reduce the number of detectors, the total search is not completely covered by detectors; if the number is increased, overlapping of detectors with self data occurs resulting in decreased detection accuracy due to false negatives. Table 2 shows the detection accuracies for each combination of hidden layers only for subject 1, bold values show maximum detection accuracy for all combinations of hidden layers. H1 are the number of neurons in first hidden layer and H2 in second hidden layer. The combination of 16/8 is selected with the maximum detection accuracy.

**Table 2:** Detection accuracy for subject 1.

**H1/H2**	**Max detection accuracy**	**Number of detectors**
15/6	0.4375	40
15/7	0.4861	30
15/8	0.5069	30
15/9	0.5694	40
15/10	0.6806	30
16/6	0.5694	30
16/7	0.7708	30
**16/8**	**0.8194**	**40**
16/9	0.4931	40
16/10	0.5903	30
17/6	0.4931	30
17/7	0.6181	40
17/8	0.5625	40
17/9	0.4375	40
17/10	0.4861	40
18/6	0.5833	40
18/7	0.5278	30
18/8	0.75	40
18/9	0.5347	30
18/10	0.50	30
19/6	0.5625	30
19/7	0.7986	30
19/8	0.6528	30
19/9	0.5278	40
19/10	0.7083	40

Table 3 shows the combination of number of hidden layer neurons and number of detectors for maximum detection accuracy for all nine subjects.

**Table 3:** Maximum detection accuracy for all nine subjects.

**Subject**	**H1/H2**	**Max detection accuracy**	**Number of detectors**
1	16/8	0.8194	40
2	18/10	0.7847	40
3	19/7	0.7917	30
4	15/10	0.7222	30
5	17/10	0.7361	30
6	16/7	0.7639	30
7	17/10	0.7361	40
8	18/8	0.7167	35
9	16/9	0.6528	30

##### Detector generation and optimization

As described earlier in the previous sections, the algorithm is inspired by the mechanism found in BIS. Each detector is generated in the space having the same number of dimensions as the data set. As mentioned in the above section that data is reduced to eight dimensions after using the best detection accuracy auto-encoder, the detector generated will also have the same number of dimensions. Before the detector training is started, the data is normalized to the range [0, 1].

**Figure 7:**
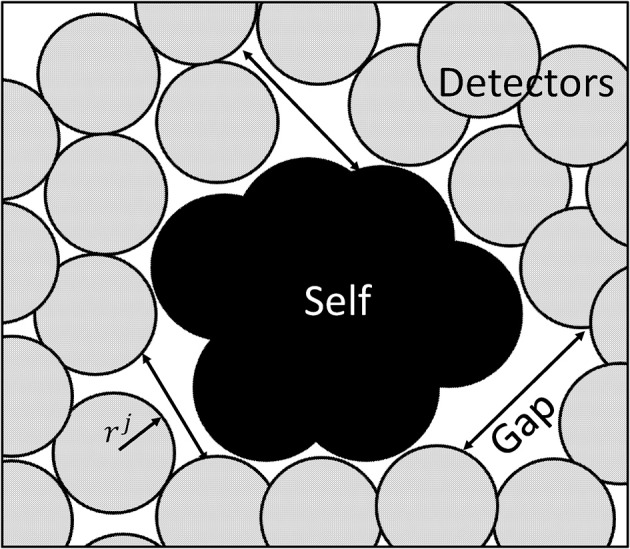
Search space with self-samples and non-self detectors.

This is done to simplify the code, which allows the radius of each detector to be defined once instead for each dimension. As suggested in Qin et al. (2008), the generation of detectors is focused on minimum number of detector yet covering all nonself space to identify and detect nonself classes. The mechanism used for detector generation takes into account the optimal detector center with maximum detector radius to influence the region having a constraint of not to detect self-matching samples. Moreover, it is also imperative that the detectors should have minimum overlap and maximum diversity factor. The maximum diversity factor will ensure coverage of the whole search space with reduced number of detectors to reduce burden of computation. Figure 7 shows the distribution of detectors in a search space.

As discussed earlier, the proposed mechanism determines the optimal detector center in a way so that the detector radius and diversity can be maximized while keeping in view the constraint of self matching detector prevention. Genetic algorithm is used to achieve the maximization and optimization of detectors under the limitation of constraint. The process is initialized with a random population of detectors in the search space, called candidate detectors. Once the population has been initialized, the training algorithm proceeds to set the radius *r*_*j*_ of the candidate detector. The set of candidate detectors is checked against the whole training set. After one complete iteration of GA, we obtain one mature optimal detector, which is stored in the optimized detector set. The implementation process of GA is repeated to obtain and store the required optimum number of the detector set (Abid et al., 2017). The pseudo code for GA is given in Table 4.

**Table 4:** Pseudo code–GA for optimization of detectors.

i = 0	set generation number to zero
begin
init_population P(0)	initialize random population of individuals (detectors)
evaluate P(0)	evaluate fitness of all initial individuals (detectors) of population while (not done) do test for termination criterion(time, fitness etc)
while (not terminated condition) Do
select P(i) from P(n-1)	select a sub-population for offspring reproduction
crossover P(i)	recombine the gene of selected parents
mutate P(i)	perturb the mated population
evaluate P(i)	evaluate its new fitness
i = i + 1	increase generation number
end

The output of training algorithm is the set of detectors (antibodies) that is used by the classification algorithm.

**Figure 8:**
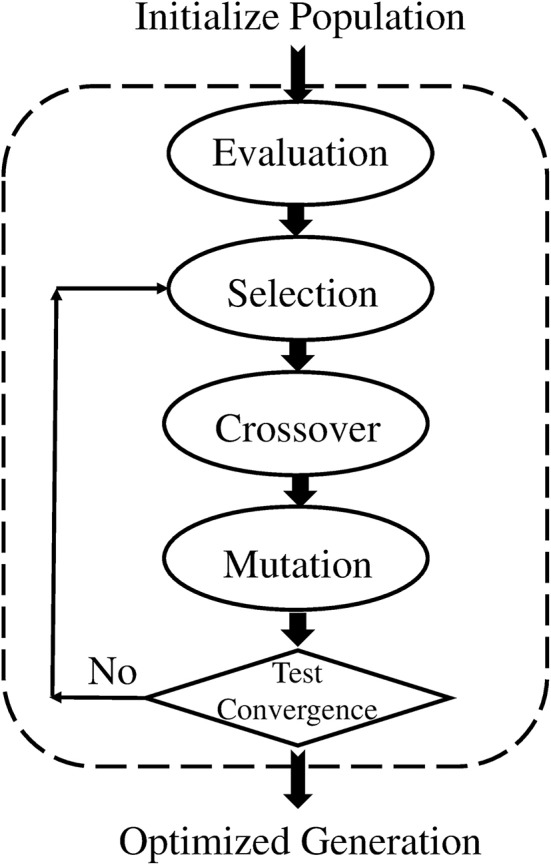
Flow diagram of GA.

The flow chart of GA is shown in Figure 8.

Abid et al. (2017) suggested the optimization of detectors using Equations 17–19. Let the set of training sample data vectors be Ω_*k*_(*k* = 1, 2, ………*K*) with self radius *R*^*k*^ = *R*^*self*^, the location of the optimal set of detectors is given by *d*_*j*_(*j* = 1, 2, ……… *N*_*d*_). Following criteria are used to maximize the detector radius *r*^*j*^. Maximization of detector radius with minimum overlapping.

(17) $$\underset{d_{j}}{\max}r^{j}=\underset{d_{j}}{\max}\left|\Omega_{k}-d_{j}\right|-R^{k}$$

Maximization of diversity factor

(18) $$f_{j}^{*}=\frac{\delta f_{j}}{s_{j}}\;where\;s_{j}=\sum_{\text{i}=1}^{D}\xi_{ij}$$

Bi-objective optimization

(19) $$\underset{d_{j}}{max}f_{j}^{*}=\underset{d_{j}}{max}\frac{\delta f_{j}}{s_{j}}=\underset{d_{j}}{max}\frac{\delta \left(\left|\Omega_{k-}d_{j}\right|-R^{k}\right)}{s_{j}},$$

where $f_{j}\text{ and }f_{j}^{*}$ are the initial fitness value and updated fitness value, respectively, *s*_*j*_ is the diversity factor and ξ_*ij*_ is defined as distance based similarity between candidate and selected detectors, and δ is the scaling factor. The proposed multi-parameter GA configuration uses Roulette wheel selection, multi-point crossover, and bit-wise mutation operator.

##### Selection of best set of optimized detectors

The selection of the best set of optimized detector set is based upon the initial and final fitness values for candidate detectors. The number of detectors to be generated is also given as an input to GA, this enables our algorithm to best fit the number of detectors according to their fitness value for complete coverage of the space. As shown in Table 2, for subject 1, the maximum accuracy is achieved with a network having combination of 16/8 and number of detectors equal to 40. The detection accuracy of self against nonself samples is calculated for a range (25–70) of number of detectors with a gap of five between the two numbers.

##### Classification of test data set

The optimized detector sets are generated, one set for each class of movement for each subject. In this way, there will be four sets of optimized mature detectors for classification of four classes. The negative selection classification algorithm (NSCA) is used for classification of four human limb movements with the help of EEG signals. The decision of class is done on the basis of the Euclidean distance of all the detectors in a set with the input test sample. The comparison rule is given in Equation (20).

(20) $$d(u,v)=\sqrt{\sum_{i=1}^{n}{\left(u_{i}-v_{i}\right)}^{2}},$$

where *u and v* are two vectors and *d* is the Euclidean distance between the vectors. Each vector of the detector set is compared for the affinity measure (Euclidean distance) with the input vector (test sample), if a match is found (*d*<*threshold*) between the test sample and any of the vector of detector set, the sample is labeled as the nonself class. In other words, a particular set of detectors trained for detection of a certain class will predict the particular class on the basis of the affinity measure rule.

The summary of our proposed methodology for the classification can be given as follows: stage 1) multi-domain feature extraction; stage 2) selection of best feature on the basis of wrapper method; stage 3) data dimensionality reduction using auto-encoder on the basis of detection accuracy; stage 4) detector generation and optimization using GA for each class; and stage 5) multi-class classification of four human limb movements using NSCA.

## Results and discussion

In this study, motor imagery EEG data pertaining to all four classes of human limb movements has been used for classification. This is an open source data set (BCI IV–Graz dataset 2a, comprising 9 subjects) widely used by researchers. Multi-domain feature extraction followed by selection of feature on the basis of wrapper method is carried out as discussed in section II–Feature Selection. In addition, MFCCs have been selected as features for further processing and classification. Bio-inspired AIS has been used for the classification of limb movements. We have used hold out validation for training of classifier with 70% training and 30% validation data. Our proposed NSCA has been applied on test data of all the subjects for the calculation of classification accuracy. Confusion matrix is used as a measure of calculation of classification accuracy of the test sample by a trained classifier. Classification accuracy has been calculated using Equation (21)

(21) $$Classification\;Accuracy=\frac{Number\;of\;correct\;predictions}{Total\;number\;of\;predictons}$$

Confusion matrix of subject 1 with classification accuracy (85.0%) is shown in Table 5. True positives (correctly classified/predicted test samples) are the diagonal of confusion matrix, and elements other than the diagonal are incorrectly classified/predicted samples.

**Table 5:** Confusion matrix of classification accuracy–subject 1.

	**Class**	**Predicted class by NSCA**			
		**Left hand**	**Right hand**	**Both feet**	**Tongue**
**True class**	Left hand	19	–	1	–
	Right hand	–	17	2	1
	Both feet	2	–	15	3
	Tongue	2	2	–	16

**Figure 9:**
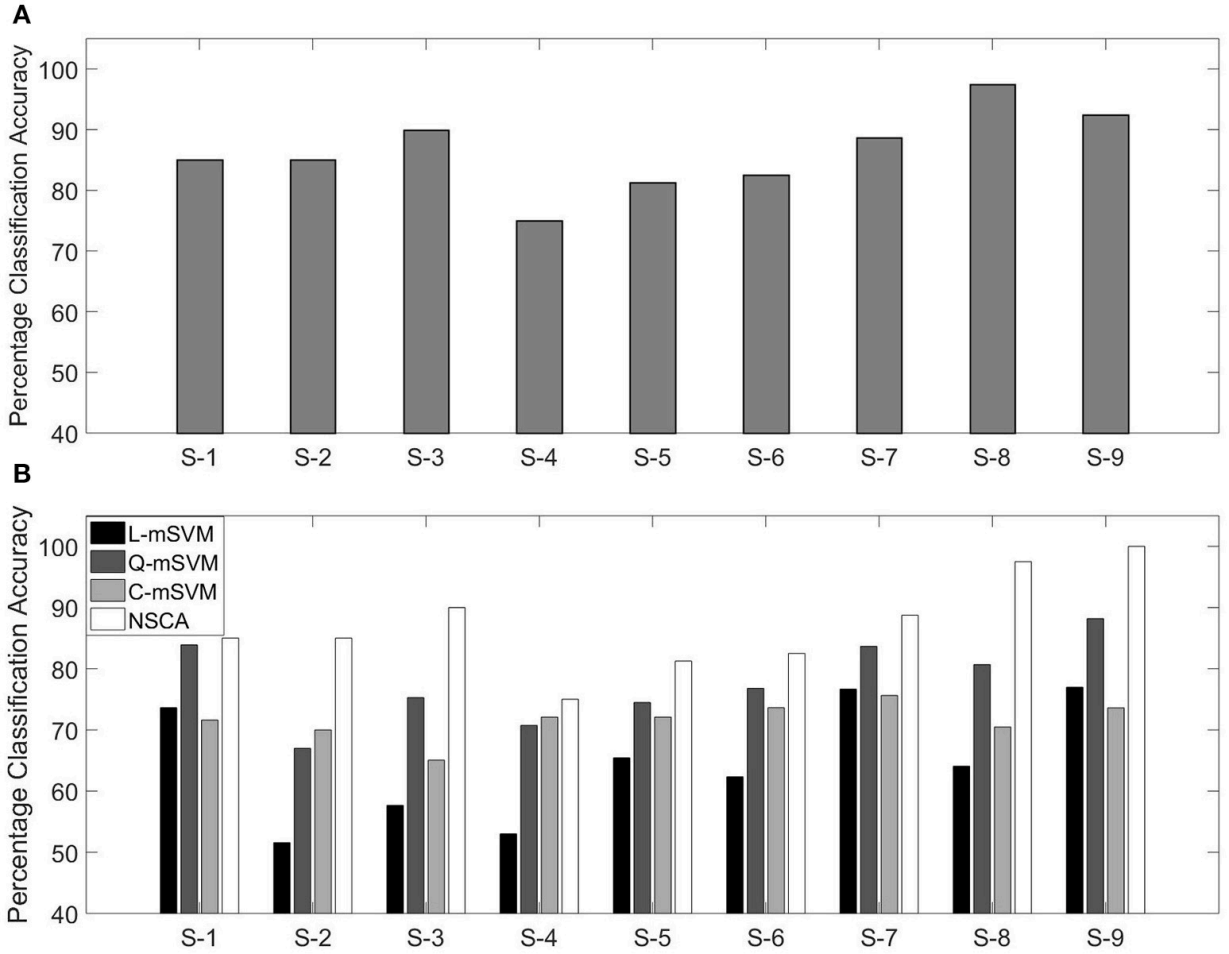
**(A)** Percentage classification accuracy using NSCA and **(B)** comparison with variants of SVM for nine subjects.

Classification accuracies using NSCA for all nine subjects are shown in Figure 9A.

In our previous research, we have used the same data for calculating the classification accuracy. We used the same feature as in this research (MFCC) as feature extraction technique, and three variants of the multi-class support vector machine (mSVM) were used for determining the classification accuracy. The three variants of mSVM were linear-mSVM (L-mSVM), quadratic-mSVM (Q-mSVM), and cubic-mSVM (C-mSVM). The results of the three variants in comparison with this research are shown in Figure 9B.

Figure 9B shows that NSCA performance for determining classification accuracy has improved as compared with our previous research. The increased performance can be seen in all nine subjects. This gives rise to the fact that NCSA as a multi-class classifier has outperformed the earlier three variants of mSVM.

A number of researchers have used the same data set as Brunner et al. (2008) for determining classification accuracy by using different feature extraction and classification techniques. In 2008, Grosse-Wentrup and Buss (Grosse-Wentrup and Buss, 2008) used SVM as a classifier in combination with the multiclass common spatial pattern (mCSP) as the feature extraction method. In 2013, Zhang et al. (2013) used a combination of complex CSP as the feature extraction technique and SVM as the classifier. In 2018, Meisheri et al. (2018) used the classification approach with mCSP and adaptive learning classifier, given a name as self-regulated interval type-2 neuro-fuzzy inference system (SRIT2NFIS). Results of all the studies mentioned above, along with our previous and current research are shown in Table 6. Bold values show maximum mean classification accuracy and minimum standard deviation for our research methodologies as compared with state of the art techniques.

**Table 6:** Classification accuracy of proposed approach as compared with other approaches.

**BCI competition data set-2008 Graz data set A**						
**Classification accuracy in %**						
**Subjects**	**Grosse-Wentrup and Buss (****2008****)**	**Zhang et al. (****2013****)**	**Meisheri et al. (****2018****)**	**Meisheri et al. (****2018****)**	**Our previous research**	**Proposed methodology**
	**mCSP with SVM**	**ComplexCSP with SVM**	**mCSP with SVM**	**mCSP with SRIT2NFIS**	**MFCC with Q-mSVM**	**MFCC with NSCA**
1	48.1	61.5	68.75	74.65	83.91	85.00
2	27.3	32.1	41.67	45.48	66.99	85.00
3	70.6	68.6	66.31	74.31	75.27	90.00
4	21.4	27.1	37.98	39.58	70.74	75.00
5	22.7	34.3	25	32.99	79.49	81.25
6	32.4	35.3	36.62	37.9	76.80	82.50
7	52.3	48	52.97	54.17	83.66	88.75
8	65.8	65.6	65.55	66.32	80.69	97.50
9	34.2	41.8	64.58	66.31	88.19	92.50
Mean	41.64	46.01	51.04	54.63	**78.48**	**86.39**
S.D	18.34	15.65	16.15	16.27	**6.72**	**6.67**

Our approach exhibits comparatively best results with state of the art techniques and our previous technique as well (which has also surpassed the state of the art techniques). Our recent technique presented in this paper has been able to differentiate among four classes of data with greater accuracy. The advantage of using GA optimized AIS NSCA is that it only needs information about one class during training of detectors. Detectors are trained for all the four classes, and these detector sets do not need to have the information of all four classes rather require only one. The optimization of the region of influence of detectors (detector radius) has resulted in coverage of space with minimum number of detectors, yet producing best results in comparison. The feature reduction technique has resulted in reduced computational expense during training and also performs better during testing.

The selection of features using the wrapper method during training has enabled our classifier to know the best feature depending upon the classification accuracy, and this has resulted in improved classification accuracy during the testing phase. As the set of detectors are optimized and saved during training, it takes less time as compared with k-fold cross validation accuracy determining methods. Moreover, owing to the fact that we are considering the short duration power spectrum of a signal using mel frequency cepstrum (MFC), performance of the EEG classification approach depends on the number of MFC coefficients. Our results are based on 12 MFC coefficients. In an EEG signal, the movements of limbs and organs produce certain frequencies patterns at the motor cortex. Our feature extraction technique alongwith NSCA has been able to differentiate the patterns of all the four classes effectively and resulted in increased classification accuracy.

It is significant to mention that the classical and powerful classification algorithm need to have the information about all the data labeled for training, however, our framework can detect the class in a binary fashion first and then can be converted into a multiclass classifier by training set of detectors on other classes as well. As mentioned and shown in our research, the data under consideration for classification is very complex and classes overlap as seen in the 2D plot. However, having the information of one class at a time, NSCA has been successfully able to classify the human limb movements with higher accuracies as compared with other recent research methodologies.

## Conclusion and future work

This paper presents a novel classification approach for the classification of complex EEG brain signals. Brain signals are called complex as these signals not only vary from subject to subject for the same limb movement but also vary within a subject at different time points of data recordings. These signal patterns change if the subject is tired or exhausted or even he/she is having mood swings. Owing to diversity, the correct classification becomes difficult but at the same time challenging as well. Our GA based optimized AIS NSCA has been able to successfully overcome the challenge as compared with the latest techniques used by researchers. The proposed framework consists of stage 1) multi-domain feature extraction; stage 2) selection of the best feature on the basis of the wrapper method; stage 3) data dimensionality reduction using auto-encoder on the basis of detection accuracy; stage 4) detector generation and optimization using GA for each class; and stage 5) multi-class classification of four human limb movements using NSCA. Results show a significant improvement in the classification accuracy as compared with the latest research. In addition, NSCA has shown an average classification accuracy of 86.39% and maximum classification accuracy of 97.50% for subject number eight.

Toward the future work, the research should dive more into the human immune system and replicate the response of the system in the field of artificial intelligence. For example, clonal selection occurs when a B-lymphocyte encounters an antigen. Antigen selects the few B-lymphocytes, out of many millions, that have cell surface antibody that best “fits” the antigen.

## Author contributions

All authors listed have made a substantial, direct, and intellectual contribution to the work and approved it for publication.

### Conflict of interest statement

The authors declare that the research was conducted in the absence of any commercial or financial relationships that could be construed as a potential conflict of interest.
